# Neutrophil extracellular traps promote macrophage pyroptosis in sepsis

**DOI:** 10.1038/s41419-018-0538-5

**Published:** 2018-05-22

**Authors:** Linsong Chen, Yanfeng Zhao, Dengming Lai, Peng Zhang, Yang Yang, Yuehua Li, Ke Fei, Gening Jiang, Jie Fan

**Affiliations:** 1 Department of Thoracic Surgery, Shanghai Pulmonary Hospital, Tongji University School of Medicine, Shanghai, 200433 China; 2 Department of Surgery, University of Pittsburgh School of Medicine, Pittsburgh,, PA 15213 USA; 3 Department of Thoracic and Cardiovascular Surgery, The Children’s Hospital of Zhejiang University School of Medicine, Hangzhou, Zhejiang China 310052; 4 Research and Development, Veterans Affairs Pittsburgh Healthcare System, Pittsburgh, PA 15240 USA; 5 McGowan Institute for Regenerative Medicine, University of Pittsburgh, Pittsburgh, PA 15219 USA

## Abstract

In response to infection, polymorphonuclear neutrophils (PMN) are recruited in the infectious sites, and employ three major strategies to fight against the microbes including phagocytosis, degranulation, and neutrophil extracellular traps (NETs). NETs are a meshwork of chromatin fibers mixed with granule-derived antimicrobial peptides and enzymes, which trap and kill the bacteria extracellularly. In this study, by using a mouse sepsis model, we identified a novel mechanism by which NETs induce macrophage (Mϕ) pyroptosis, a caspase-1-dependent regulated cell death. We show that NET-derived HMGB1, acting through RAGE and dynamin-dependent signaling, triggers an intra-Mϕ cascade of molecular events including cathepsin B (CatB) release from the ruptured lysosomes, followed by pyroptosome formation and caspase-1 activation, and subsequent Mϕ pyroptosis. The study further demonstrates that Mϕ pyroptosis augments inflammatory responses following sepsis. These findings shed light on the proinflammatory role of NETs in mediating PMN–Mϕ interaction, which therefore influences the progress of inflammation following infection.

## Introduction

Sepsis, a leading cause of human death worldwide, is characterized by excessive inflammation in response to infection^1^. During septic peritonitis, polymorphonuclear neutrophils (PMN) and monocytes are recruited in the peritoneal cavity, and the latter differentiate into inflammatory macrophages (Mϕ)^2^. The interaction between PMN and Mϕ has been suggested as an important factor that regulates inflammation following trauma, hemorrhagic shock, and endotoxemia, as well as other pathological conditions^3–7^. For instance, we have reported that exosomes released from the hemorrhagic shock-activated alveolar Mϕ promote PMN necroptosis in the lungs^5^. Others have demonstrated that neutrophil extracellular traps (NETs) acting through inducing cytokine production from Mϕ promote inflammation and the development of atherosclerosis^7^. However, in sepsis, the role of the interaction between PMN and Mϕ in the development of inflammation and the underlying mechanism remains unclear.

In response to microbial infection, one of the defensing mechanisms of the host is to release nuclear contents of the PMN into the extracellular space to trap and kill the microbes, known as NETs. Since first discovered in 2004^8^, NETs have been recognized as an important strategy of the host immune system to respond against infections^9^. NETs are composed of decondensed chromatin decorated with granular and cytoplasmic proteins, which may serve as danger-associated molecular patterns (DAMPs) playing critical role in the progression of host inflammation^10,11^.

High-mobility group box 1 (HMGB1), a highly conserved nuclear protein widely present in the nucleus and cytoplasm of nearly all cell types, is the prototypic DAMP molecule when released into the extracellular space^12^. Our previous studies reported that HMGB1 plays a critical role in inducing pyroptosis of Mϕ and vascular endothelial cell (EC)^13,14^. Pyroptosis is a caspase-1-dependent form of regulated cell death, which is usually triggered by various pathological stimuli, i.e., intracellular pathogens and extracytoplasmic stimuli^15^. The characteristics of pyroptosis include cell swelling, rapid plasma membrane rupture, and release of proinflammatory contents^16^. Pyroptotic cell-released danger signals or DAMP molecules enhance inflammatory responses^17^.

In this study, we identified a novel mechanism of PMN–Mϕ interaction in sepsis. We demonstrate in the mouse cecal ligation and puncture (CLP) sepsis model that PMN through ejecting NETs induce peritoneal Mϕ pyroptosis. We further confirm that HMGB1 released from NETs acting through the receptor for advanced glycation end products (RAGE) initiates dynamin-dependent signaling pathway that includes cathepsin B (CatB) activation, pyroptosome formation, and caspase-1 activation, which in turn leads to Mϕ pyroptosis. These findings suggest a previously unidentified pathway of PMN–Mϕ cross-talk, which causes enhanced Mϕ death and subsequent exaggerated post-sepsis inflammation.

## Results

### Sepsis induces peritoneal Mϕ pyroptosis

**Fig. 1:**
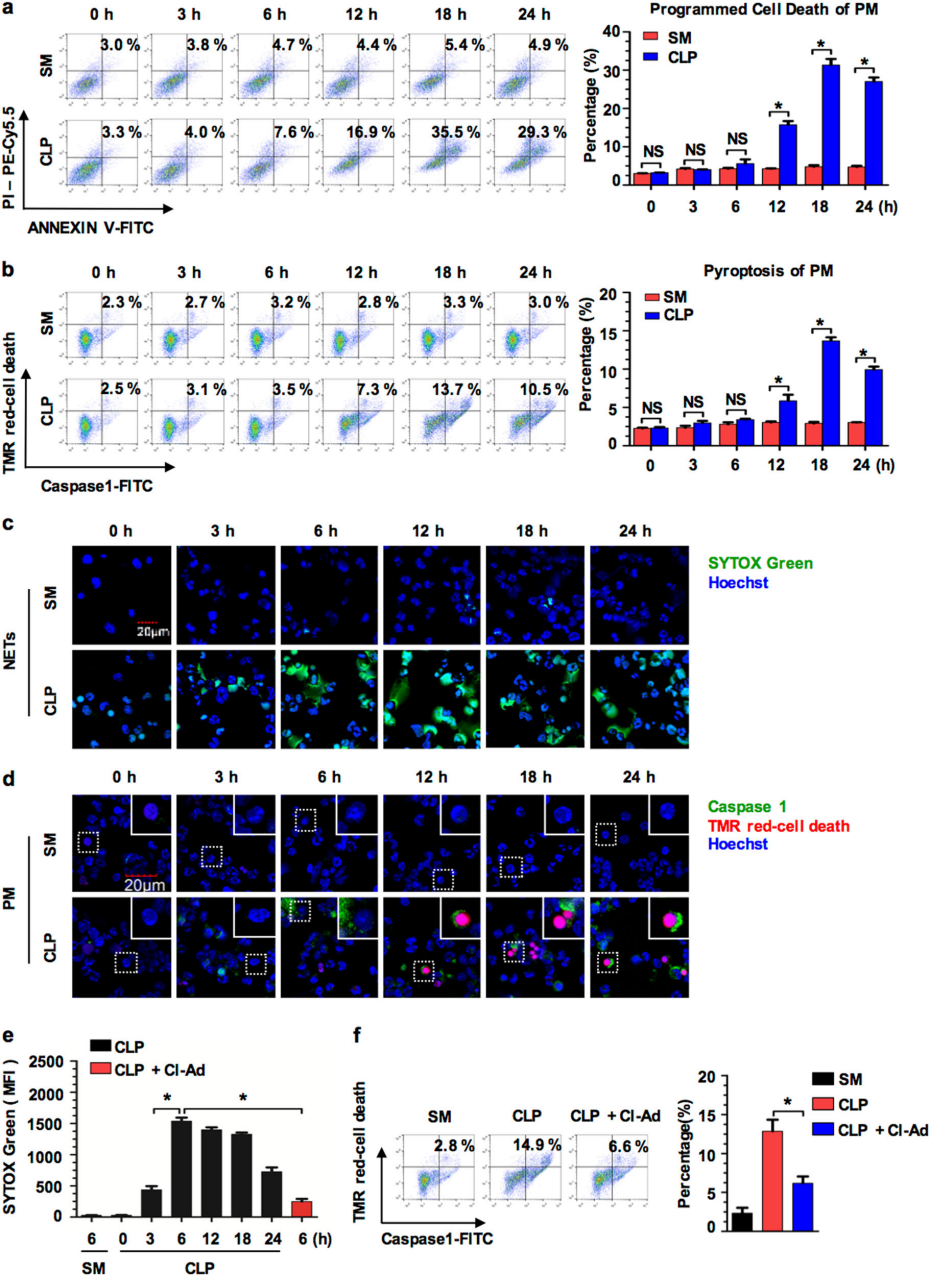
Sepsis induces peritoneal Mϕ (PMϕ) pyroptosis. **a**–**c** Mice were subjected to CLP for up to 24 h. **a** Regulated PMϕ death was assessed by PE-Annexin-V and 7-AAD double-staining using flow cytometry. **b** The pyroptosis of PMϕ was assessed by Cell Death Reagent-TMR and Alexa Fluor 488-labeled caspase-1 FLICA double-staining by flow cytometry. **c**, **d** Peritoneal lavage fluid (PLF) was collected from mice that were subjected to either CLP or sham for 0, 3, 6, 12, 18, and 24 h, and then each of the PLF samples was divided into two aliquots for florescence microscopy to determine NETs (**c**) and macrophage pyroptosis (**d**), respectively. NET formation in the supernatant of PLF was assessed by staining with DNA-binding dye, SYTOX Green. PMϕ were stained with Cell Death Reagent-TMR (red) and Alexa Fluor 488-labeled caspase-1 FLICA (green); the double-stained pyroptotic cells were detected by confocal microscopy. **e** Mice were subjected to CLP for up to 24 h, and Cl-Ad (50 mg/kg B.W.) in some experiments was injected into the peritoneal cavity at 30 min prior to CLP. Quantification of NET formation in the supernatant of PLF was assessed by staining with DNA-binding dye, SYTOX Green. **f** Mice were subjected to CLP for 18 h with or without Cl-Ad pre-treatment. The pyroptosis of PMϕ was assessed by Cell Death Reagent-TMR and Alexa Fluor 488-labeled caspase-1 FLICA double-staining by flow cytometry. All results are representative of the five independent experiments, and graphs depict the values of the mean and the S.E.M. *P < 0.05, as compared between two groups. NS, no significant difference. Higher magnification images for the selected area are shown in the boxed insets (original magnification ×600)

In CLP-induced sepsis, we found that a considerable part of the peritoneal Mϕ (PMϕ) underwent cell death. As shown in Fig. 1, mice subjected to CLP exhibited a gradual increase in PMϕ death. The PMϕ death, as defined as double staining of Annexing V and 7-AAD, reached a peak (~36%) at 18 h after CLP. In order to determine the type of PMϕ death, PMϕ collected from the peritoneal lavage fluid (PLF) were further detected for nuclear fragmentation, caspase-1 activation, and the characteristics of pyroptosis, by staining the cells with TMR-Cell Death Reagent and Alexa Flour 488-labeled caspase-1 FLICA, and measured by flow cytometry. We observed ~14% pyroptotic PMϕ at 18 h after CLP (Fig. 1b). These results suggest that pyroptosis is responsible for ~39% of the PMϕ death following sepsis.

Interestingly, we further observed NET formation in PLF, associated with the exhibition of PMϕ pyroptosis following CLP. PLF samples were collected from mice that were subjected to either CLP or sham operation for 0 to 24 h, and each of the PLF samples was divided into two aliquots for florescence microscopy of NETs and PMϕ pyroptosis, respectively. Using SYTOX green staining of DNA and florescence microscopy, we detected in PLF a progressive increase in NET formation, which started at as early as 3 h and reached a peak at 6 h after CLP, and remained at a high level for at least 24 h (Fig. 1c, e). Associated with the formation of NETs, PMϕ pyroptosis occurrence starts at 12 h after CLP (Fig. 1d). Intraperitoneal injection (i.p.) of Cl-Amidine (Cl-Ad, 50 mg/kg B.W.), an inhibitor of NET formation by suppressing peptidylarginine deiminase 4 (PAD4)^18,19^, at 30 min prior to CLP prevented NET formation following sepsis (Fig. 1e). Notably, the suppression of NET formation was associated with the decrease in PMϕ pyroptosis at 18 h after CLP (Fig. 1f). These results led us to determine the causal role of NETs in PMϕ pyroptosis.

### NETs induce PMϕ pyroptosis

**Fig. 2:**
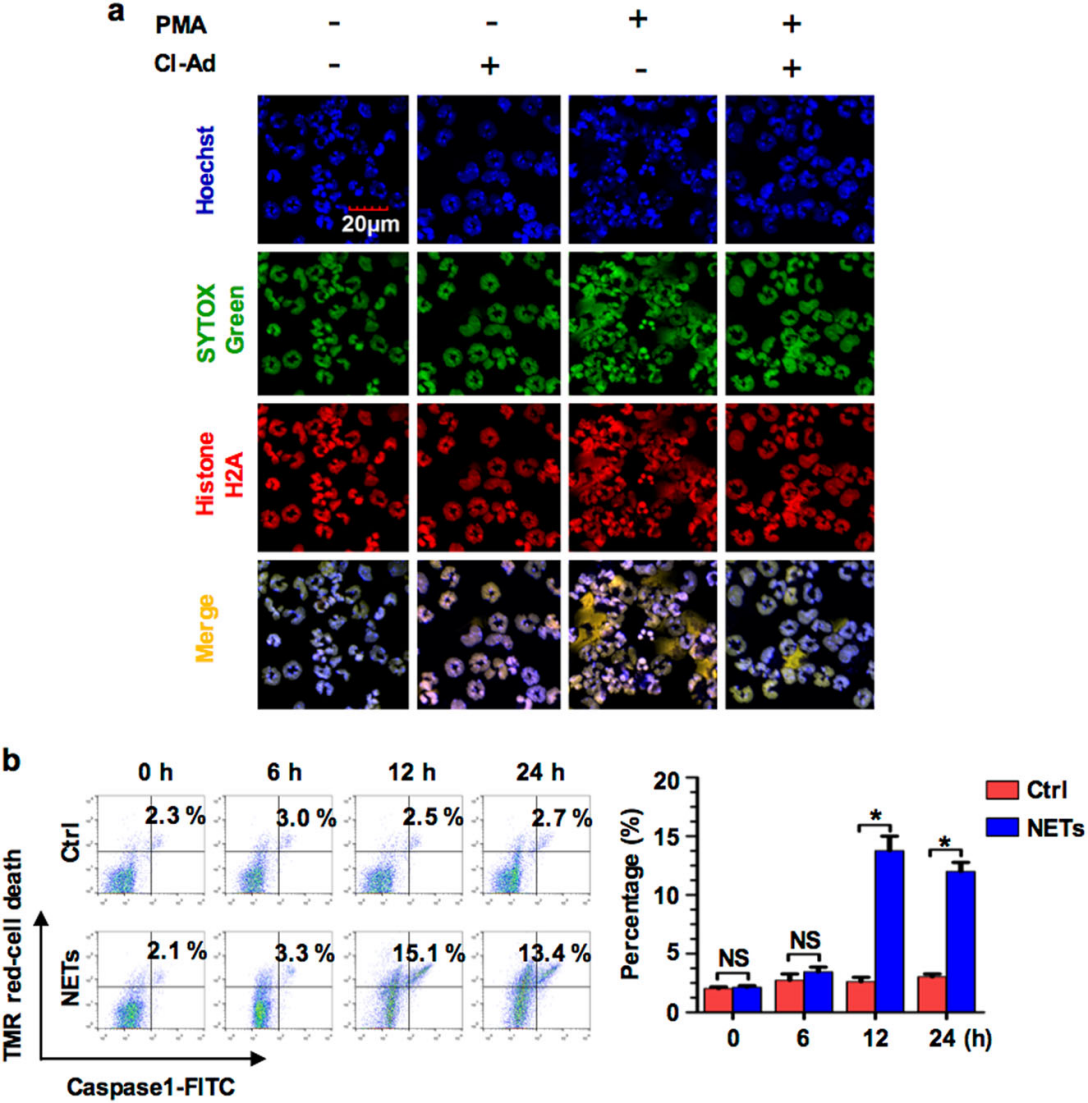
NETs induce PMϕ pyroptosis . **a** Peritoneal PMN were pretreated with or without Cl-Ad (200 μM) for 30 min, and then were stimulated by PMA (50 nM) for 2 h to induce NET formation that was confirmed by SYTOX green and histone 2A double staining, and florescence microscopy. Fluorescent images were obtained by confocal microscopy (original magnification ×600). **b** BMDM were incubated with NETs for up to 24 h. BMDM pyroptosis was assessed by Cell Death Reagent-TMR and Alexa Fluor 488-labeled caspase-1 FLICA by flow cytometry. All the results are representative of the five independent experiments, and graphs depict the values of the mean and the S.E.M. *P < 0.05, compared between two groups. NS, no significant difference

To investigate a direct role of NETs in regulating Mϕ death, we collected peritoneal PMN, which were recruited into the peritoneal cavity in response to i.p. injection of 9% casein, and stimulated the PMN in vitro with PMA (50 nM) for 2 h to induce NETs^20^. The NET formation was confirmed by SYTOX green and histone double staining, and florescence microscopy, as shown in Fig. 2a. The induced NETs in the culture medium supernatant were then co-cultured with normal bone marrow-derived macrophages (BMDM) for up to 24 h, followed by the detection of BMDM pyroptosis. As shown in Fig. 2b, NETs induced BMDM pyroptosis, which reached a peak at 12 h after the co-culture; however, the medium supernatant from unstimulated PMN did not cause BMDM pyroptosis.

These results suggest that NET formation in the peritoneal cavity following sepsis is responsible for PMϕ pyroptosis.

### NET-derived HMGB1 induces PMϕ pyroptosis

**Fig. 3:**
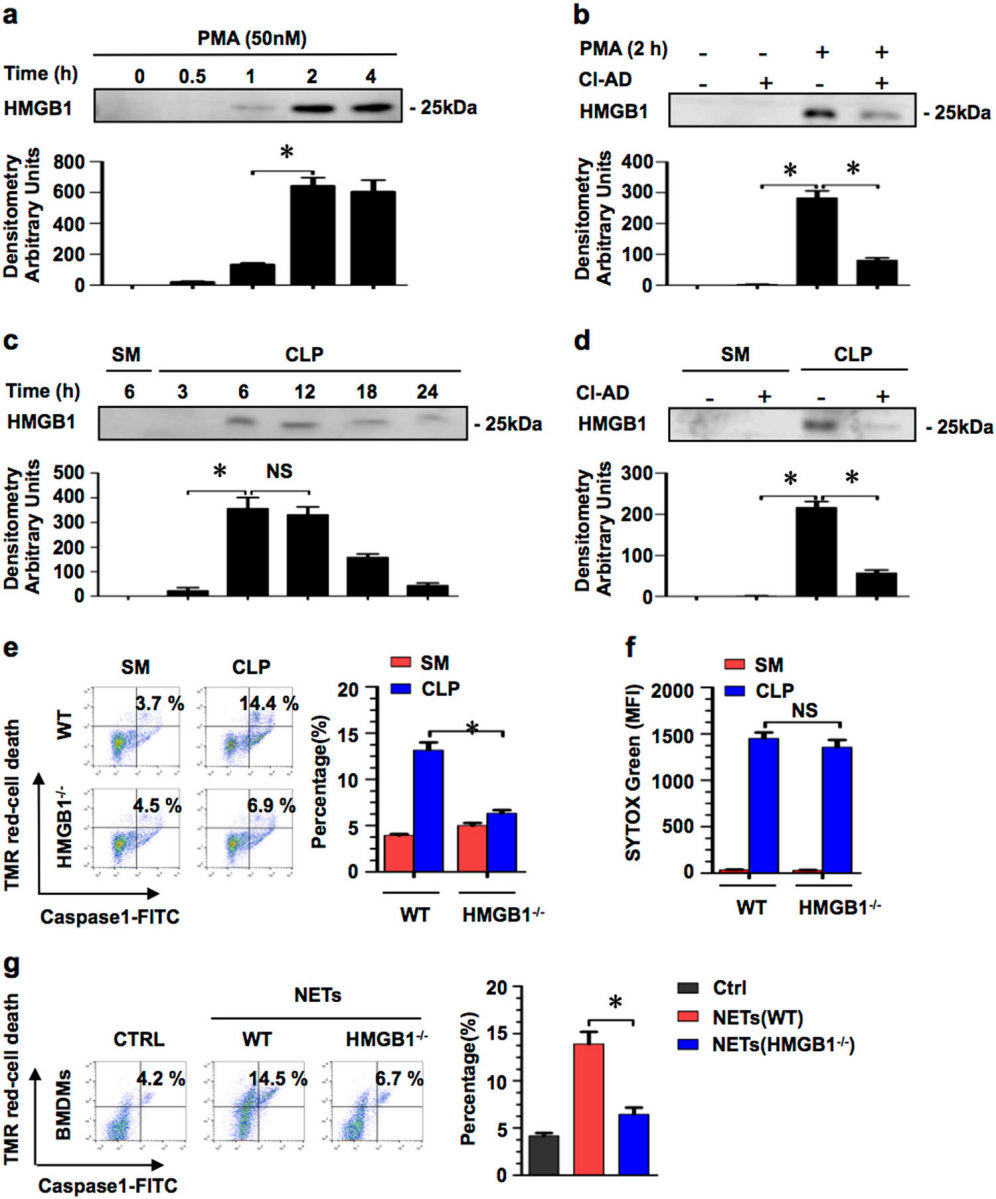
NET-derived HMGB1 induces PMϕ pyroptosis. **a** Peritoneal PMN were stimulated with PMA (50 nM) for up to 4 h to induce NETs, and HMGB1 in the medium supernatant was measured by western blotting. **b** Peritoneal PMNs stimulated with PMA (50 nM) for 2 h with or without Cl-Ad (200 μM). HMGB1 was measured in the medium supernatant by western blotting. **c** Mice were subjected to CLP for up to 24 h, and HMGB1 was measured in the PLF supernatant by western blotting. **d** Mice were subjected to CLP for 6 h with or without i.p. injection of Cl-Ad (50 mg/kg B.W.), and HMGB1 was measured in the PLF supernatant by western blotting. **e** WT and HMGB1^−/−^ mice were subjected to CLP for 18 h, and then PMϕ pyroptosis was assessed by Cell Death Reagent-TMR and Alexa Fluor 488-labeled caspase-1 FLICA double-staining by flow cytometry. **f** WT and HMGB1^−/−^ mice were subjected to CLP for 6 h. Quantification of NET formation in the supernatant of PLF was assessed by SYTOX Green. **g** NETs induced from WT and HMGB1^−/−^ PMN were co-cultured with WT normal BMDM for 12 h. BMDM pyroptosis was assessed by Cell Death Reagent-TMR and Alexa Fluor 488-labeled caspase-1 FLICA double-staining by flow cytometry. All the results are representative of the five independent experiments, and graphs depict the values of the mean and the S.E.M. *P < 0.05, compared between the two groups. NS, no significant difference

Our previous studies showed that HMGB1 can induce cell pyroptosis^13,14^, which led us to hypothesize that HMGB1 released from the NETs might serve as a critical mediator to induce PMϕ pyroptosis, as considering that NETs consist of decondensed chromatin, and HMGB1 is one of the chromatin proteins. We first determined whether the NET component includes HMGB1. Peritoneal PMN were isolated and stimulated with PMA (50 nM) for up to 4 h to induce NET formation in the presence and absence of NET inhibitor Cl-Ad (200 μM)^21^, and then HMGB1 in the PMN medium supernatant was measured by western blotting. As shown in Fig. 3a, b, at 2 h after PMA stimulation, with NET formation, HMGB1 started to increase in the supernatant; however, the NET inhibitor Cl-Ad prevented HMGB1 increase in the supernatant. These in vitro observations were recapitulated in vivo. HMGB1 level in PLF was significantly elevated at 6 h after CLP, and maintained at a high level for at least 24 h (Fig. 3c). This elevation in the HMGB1 level was suppressed by a pre-treatment with i.p. injection of NET inhibitor Cl-Ad (Fig. 3d). These results suggest that NET formation associates with HMGB1 release.

To determine whether the NET-derived HMGB1 induces PMϕ pyroptosis, we subjected wild-type (WT) and HMGB1^−/−^ mice to CLP for 18 h, and then measured PMϕ pyroptosis. HMGB1 deficiency significantly decreased the PMϕ pyroptosis, as compared to that in the WT mice (Fig. 3e), although NET formation in the CLP groups was not affected by HMGB1 deficiency (Fig. 3f). To specifically define the role of the NET-derived HMGB1 in inducing Mϕ pyroptosis, we induced NETs in WT and HMGB1^−/−^ PMN in vitro and co-cultured the NETs with WT normal BMDM for 12 h. As shown in Fig. 3g, HMGB1 deficiency significantly attenuates the NET-induced BMDM pyroptosis, as compared to the group treated with WT NETs. These results demonstrate an important role of NET-released HMGB1 in inducing Mϕ pyroptosis.

### RAGE–dynamin signaling mediates NET-induced PMϕ pyroptosis

**Fig. 4:**
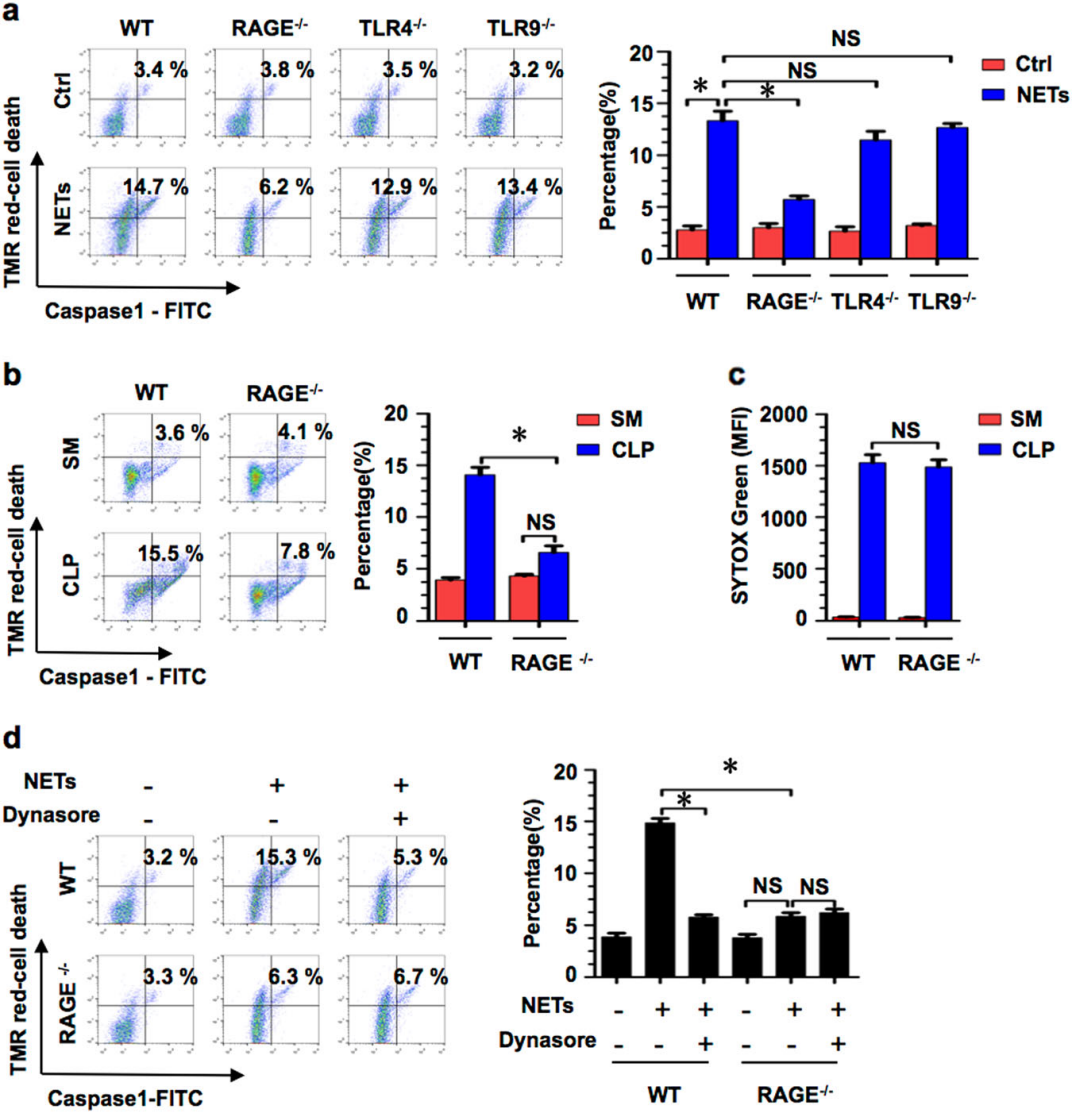
RAGE–dynamin signaling mediates NET-induced PMϕ pyroptosis. **a** BMDM derived from WT, RAGE^−/−^, TLR4^−/−^, and TLR9^−/−^ mice were stimulated by NETs for 12 h. BMDM pyroptosis was assessed by Cell Death Reagent-TMR and Alexa Fluor 488-labeled caspase-1 FLICA double-staining by flow cytometry. **b** WT and RAGE^−/−^ mice were subjected to CLP for 18 h and the PMϕ pyroptosis was assessed by Cell Death Reagent-TMR and Alexa Fluor 488-labeled caspase-1 FLICA double-staining by flow cytometry. **c** WT and RAGE^−/−^ mice were subjected to CLP for 6 h, and then the quantification of NETs in the PLF supernatant was measured by the mean fluorescence intensity (MFI) of SYTOX green. **d** BMDM derived from WT and RAGE^−/−^ mice were stimulated by NETs with or without dynasore (30 μg/ml), and BMDM pyroptosis was assessed by Cell Death Reagent-TMR and Alexa Fluor 488-labeled caspase-1 FLICA double-staining by flow cytometry. All the results are representative of the five independent experiments, and graphs depict the values of the mean and the S.E.M. *P < 0.05, compared between the two groups. NS, no significant difference

To determine whether NET-induced Mϕ pyroptosis following sepsis is a specific receptor-dependent event, we isolated BMDM from WT, RAGE^−/−^, TLR4^−/−^, and TLR9^−/−^ mice, and then treated the cells with NETs (or the medium supernatant of the unstimulated PMN as a control) for 12 h. The results showed that RAGE deficiency effectively prevented Mϕ pyroptosis induced by NETs, whereas the genetic deletion of TLR4 or TLR9 failed to block Mϕ pyroptosis (Fig. 4a). We further subjected RAGE^−/−^ mice to CLP and observed that RAGE deficiency significantly prevented PMϕ pyroptosis at 18 h after CLP, as compared to that in the WT mice (Fig. 4b), although there was no significant change in NET formation between these two groups (Fig. 4c).

Our previous in vitro study has shown that RAGE–dynamin signaling mediates HMGB1-induced Mϕ pyroptosis^13^. However, this mechanism has yet to be determined in an in vivo sepsis model. To elucidate the role of RAGE–dynamin signaling in NET-induced PMϕ pyroptosis, we applied dynamin inhibitor dynasore (30 µg/ml) in the NET-BMDM co-culture system, and found that dynasore exhibited a suppressive effect on the NET-induced BMDM pyroptosis (Fig. 4d). Collectively, these results show that NET-derived HMGB1 mediates Mϕ pyroptosis via a RAGE–dynamin pathway.

### Lysosome destabilization and cathepsin B activation are required for pyroptosome formation and PMϕ pyroptosis

**Fig. 5:**
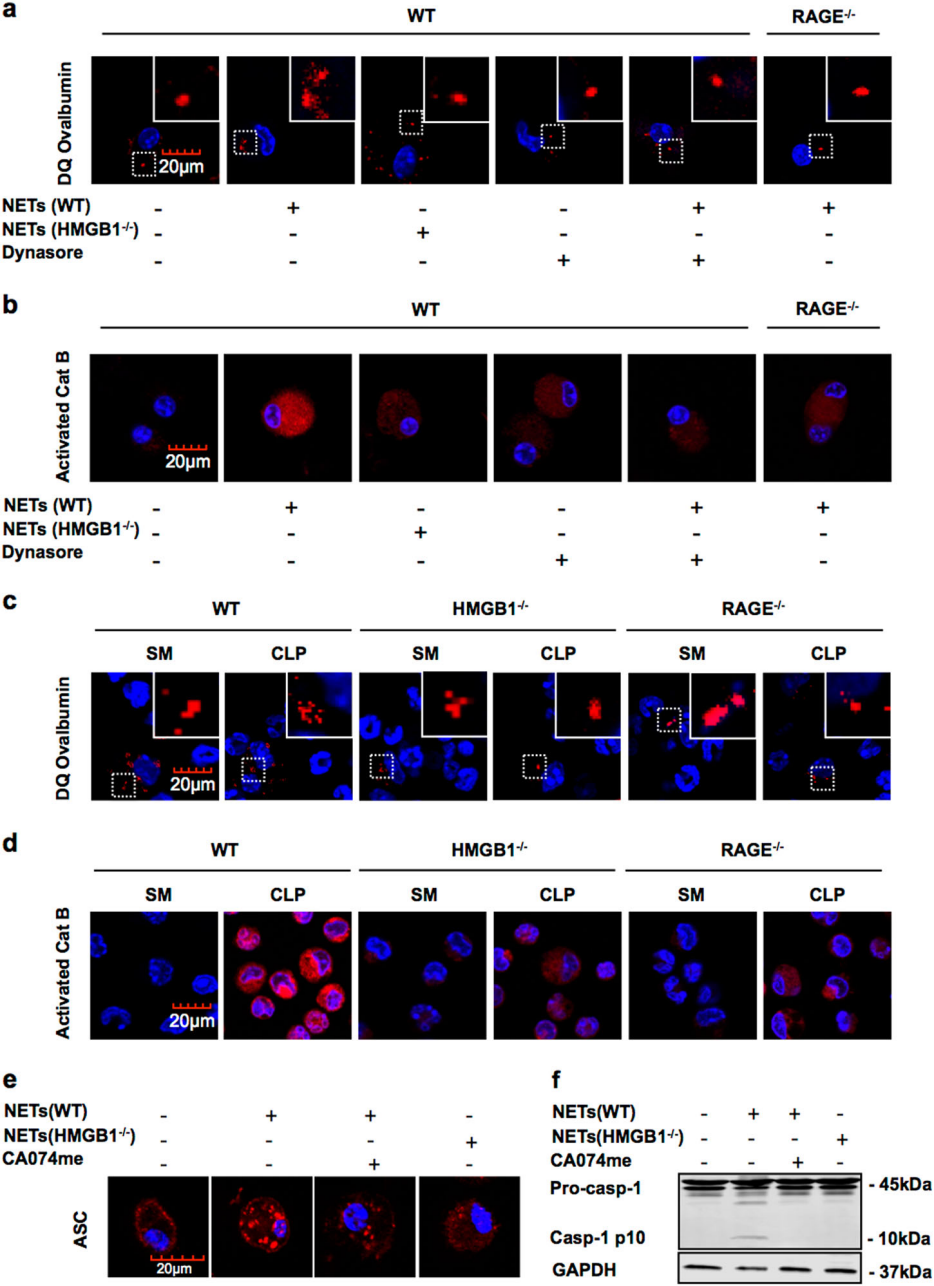
Lysosome destabilization and Cathepsin B activation are required for pyroptosome formation and PMϕ pyroptosis. **a**, **b** BMDM derived from the WT and the RAGE^−/−^ mice were stimulated by NETs derived from WT and HMGB1^−/−^ mice with or without dynasore (30 μg/ml) for 9 h. **a** The cells were incubated with DQ ovalbumin (red) for 1 h to visualize lysosome integrity by confocal microscopy. **b** The cells were stained with Magic Red CatB detection reagent (red) to visualize the activated CatB by confocal microscopy. **c**, **d** WT, HMGB1^−/−^, and RAGE^−/−^ mice were subjected to CLP for 12 h. **c** The PMϕ were incubated with DQ ovalbumin (red) for 1 h to visualize lysosome integrity. **d** PMϕ were stained with Magic Red CatB detection reagent (red) to visualize the activated CatB by confocal microscopy. **e**, **f** BMDM derived from WT mice were stimulated by NETs derived from WT and HMGB1^−/−^ mice for 9 h with or without CA-074-me (10 μM). **e** ASC foci were stained to visualize pyroptosome formation; **f** Caspase-1 cleavage (10 kDa) in the cell lysates was detected by western blotting. All the results are representative of the five independent experiments. Higher magnification images for the selected area are shown in the boxed insets (original magnification ×600)

It has been reported that pyroptosome, a complex including adaptor protein apoptosis-associated speck-like protein containing a CARD (ASC), also known as ASC focus, recruits pro-caspase-1, resulting in its activation and proteolysis of the mature form via neighboring activated caspase-1 proteins^22^. Our previous study showed that pyroptosome, rather than inflammasome, mediates HMGB1-induced Mϕ pyroptosis via lysosome enzyme cathepsin B (CatB)-dependent pathway^13^. To determine whether CatB pathway contributes to NET-induced Mϕ pyroptosis, we treated WT BMDM with NETs that were induced from WT or HMGB1^−/−^ PMN in the presence or absence of dynasore (30 µg/ml) for 9 h, followed by measuring the lysosome integrity and CatB activation in the BMDM using fluorescence-tagged DQ ovalbumin and Magic Red CatB detection reagent, respectively. The WT NETs induced lysosome rupture and CatB activation in WT Mϕ, while dynasore effectively blocked these effects (Fig. 5a, b). HMGB1^−/−^ NETs did not induce the lysosome destabilization, and CatB activation, as shown in Fig. 5a, b. In addition, WT NETs failed to induce lysosome rupture and CatB activation in RAGE deficiency Mϕ (Fig. 5a, b). Furthermore, we subjected the WT, HMGB1^−/−^, and RAGE^−/−^ mice to CLP for 12 h, and detected lysosome destabilization and CatB activation in PMϕ. As shown in Fig. 5c, d, CLP caused the lysosome rupture and CatB activation in the PMϕ from WT mice, but not in the PMϕ from the HMGB1^−/−^ and RAGE^−/−^ mice.

To determine whether pyroptosome formation is implicated in the Mϕ pyroptosis induced by NET-derived HMGB1, we stimulated WT BMDM with WT NETs or HMGB1^−/−^ NETs in the presence or absence of CatB inhibitor CA-074-me (10 μM)^13^, followed by visualizing the ASC focus using florescence-tagged ASC antibody and confocal microscopy, and detecting caspase-1 activation by western blotting. Figure. 5e, f shows that WT NETs induced ASC foci formation and caspase-1 activation, whereas CatB inhibitor prevented these effects of WT NETs; HMGB1^−/−^ NETs failed to induce ASC foci formation and caspase-1 activation. Taken together, these results suggest that NET-derived HMGB1 mediates Mϕ pyroptosis through RAGE–CatB–ASC–Caspase1 signaling pathway.

### NET-induced Mϕ pyroptosis enhances inflammatory responses

**Fig. 6:**
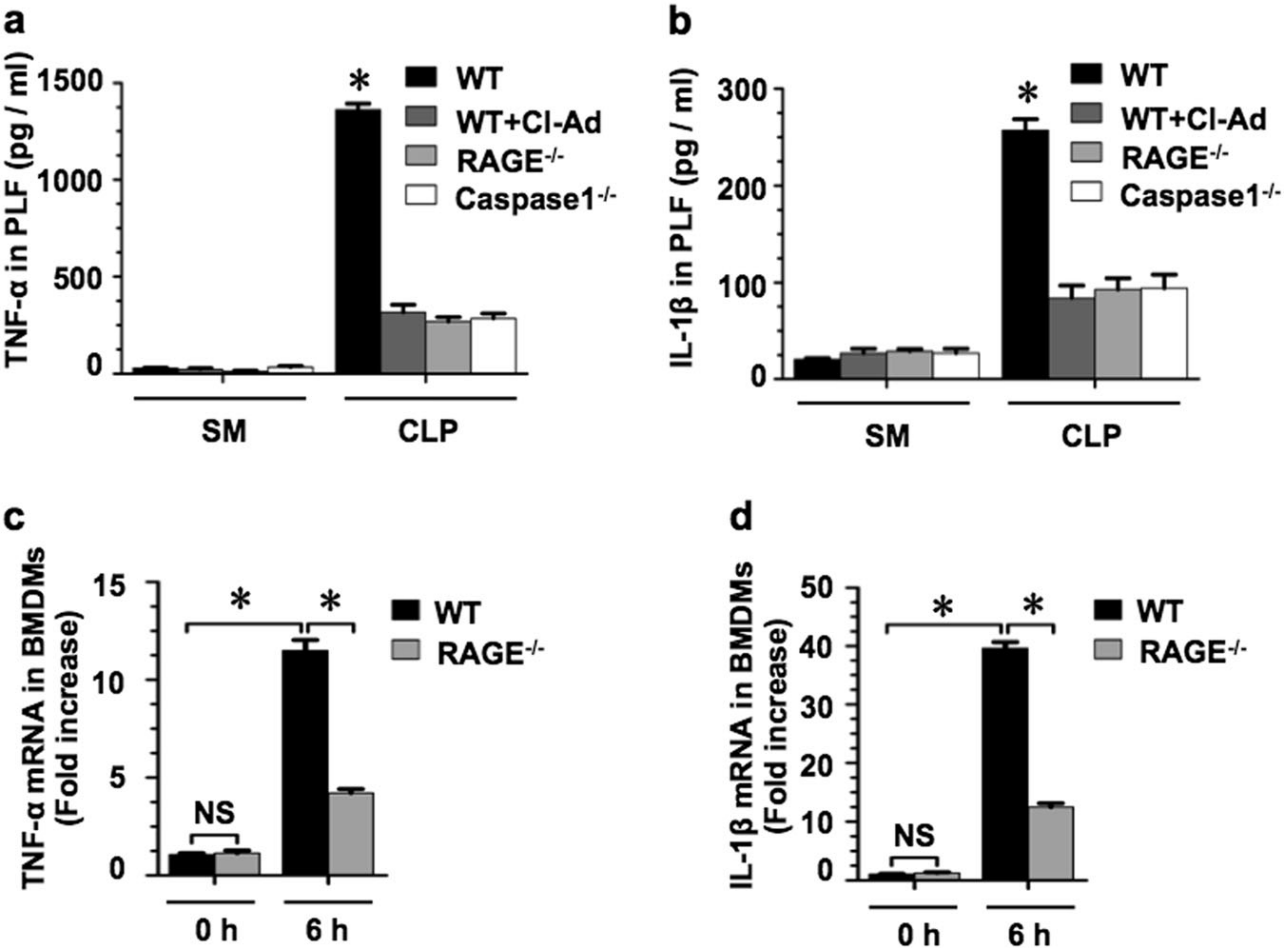
NET-induced Mϕ pyroptosis enhances inflammatory responses. **a**, **b** WT and RAGE^−/−^ mice were subjected to CLP for 18 h; the TNF-α and IL-1β levels in the PLF were measured by ELISA. **c**, **d** BMDM derived from WT and RAGE^−/−^ mice were treated with NETs for 12 h to induce pyroptosis in the upper well of the Transwell, followed by co-incubating with untreated WT BMDM, which were in the bottom well of the Transwell, for additional 6 h. TNF-α and IL-1β mRNA levels in the WT BMDM in the bottom well were then measured by qRT-PCR. The graphs depict the values of the mean and the S.E.M. *n* = 5, *P < 0.05, compared between two groups. NS, no significant difference

We have previously reported that pyroptotic EC increased the inflammatory cytokine expression in non-pyroptotic cells, suggesting a proinflammatory effect of the pyroptotic cells^14^. To determine the role of Mϕ pyroptosis in acute inflammatory response, WT, RAGE^−/−^, and caspase1^−/−^ mice were subjected to CLP for 18 h with or without i.p of NET inhibitor Cl-Ad (50 mg/kg B.W.), and then tumor necrosis factor-α (TNF-α) and the interleukin-1β (IL-1β) concentrations in PLF were measured by enzyme-linked immunosorbent assay (ELISA). As shown in Fig. 6a, b, TNF-α and IL-1β levels significantly increased in the PLF collected from WT mice following CLP, whereas the genetic deletion of either RAGE or caspase-1 markedly decreased the cytokines release in response to CLP. Furthermore, NET inhibitor Cl-Ad suppressed the release of TNF-α and IL-1β in PLF after sepsis. In order to determine that the observed influences are specifically derived from Mϕ pyroptosis, we first treated WT BMDM and RAGE^−/−^ BMDM (as a negative control) with NETs for 12 h to induce pyroptosis, and then co-cultured the pyroptotic BMDM with normal WT BMDM in the Transwell for 6 h to assess the effect of pyroptotic Mϕ on normal neighboring Mϕ. As shown in Fig. 6c, d, WT normal Mϕ co-cultured with pyroptotic BMDM (NET-treated WT BMDM) exhibited a significant increase in TNF-α and IL-1β mRNA expression; however, WT normal Mϕ co-cultured with NET-treated RAGE^−/−^ BMDM, which were not induced to pyroptosis, presented a much lower level of TNF-α and IL-1β mRNA expression.

## Discussion

Ungoverned inflammation is responsible for high morbidity and mortality during sepsis^23^. The interaction between innate immune cell populations, especially the interaction between PMN and Mϕ, plays a critical role in the development of inflammation^24^. In this study, we demonstrate a previously unidentified role for PMN–Mϕ interaction in promoting inflammation in sepsis. We showed that while NETs function as an anti-bacteria mechanism, they can also induce Mϕ death, particularly pyroptosis, which in turn enhances inflammation.

In response to infection, PMN are recruited into the infectious sites and employ three major strategies to fight against the microbes including phagocytosis, degranulation, and NETs. NETs are a meshwork of chromatin fibers mixed with granule-derived antimicrobial peptides and enzymes, such as elastase, cathepsin G, and myeloperoxidase^8^. Accompanied with NET ejection, a range of molecules are released into the extracellular space. Some endogenous molecules serve as DAMPs and exert the role of proinflammatory cytokine in the development of inflammatory diseases including acute organ injury^7,25–28^. However, the mechanism by which NET-derived DAMPs promote inflammation, remains poorly addressed. In this study, we revealed that HMGB1 released with NETs mediates Mϕ death, particularly pyroptosis, thereby amplifying the inflammation. We demonstrate that NET formation was associated with increased extracellular level of HMGB1 and inhibition of NET formation by Cl-Ad decreased the extracellular HMGB1 in vivo and in vitro. Although an elevated basal level of HMGB1 in PLF was detected after CLP, NET-caused further increase in the HMGB1 level in PLF seems required for PMϕ pyroptosis, since inhibition of NET formation significantly decreased Mϕ pyroptosis. The role of NET-released HMGB1 in inducing Mϕ pyroptosis was further evidenced by the observation that NETs formed from HMGB1^−/−^ PMN failed to induce Mϕ pyroptosis.

As a DAMP molecule, HMGB1 implicates as an endogenous proinflammatory mediator to induce nuclear factor-kB activation and augments the secretion of TNF-α, IL-1β, IL-6, and IL-12^29–31^. Our previous studies demonstrated a novel function of HMGB1 in inducing Mϕ and vascular EC pyroptosis in non-infectious settings^13,14^. We demonstrated that HMGB1 acting through RAGE and dynamin-dependent signaling initiated HMGB1 endocytosis, which in turn induced cell pyroptosis. The endocytosis of HMGB1 triggered a cascade of molecular events including CatB release from the ruptured lysosomes, followed by pyroptosome formation and caspase-1 activation. However, it was not clear whether this pathway was also valid in sepsis. The current study not only explored the source of HMGB1 that induces Mϕ pyroptosis, but also validated the pathway in sepsis model. We demonstrate in this study that suppression of HMGB1–RAGE binding or its downstream events including RAGE deletion, inhibition of dynamin, CatB, and caspase-1 activation prevent Mϕ pyroptosis. These findings support a causal role for HMGB1–RAGE–dynamin signaling in the induction of cell pyroptosis, which is valid in both infectious and non-infectious conditions.

Pyroptosis was primarily defined as caspase-1-dependent death form^32^. Activated caspase-1 cleaves Gasdermin-D (GSDMD) to generate a mature GSDMD that induces pore formation on the membrane, resulting in cell swelling, plasma membrane rupture, and release of proinflammatory intracellular contents^33^. Release of intracellular molecules from the pyroptotic cells into the extracellular space is thought to be a proinflammatory event, which would result in exaggerated inflammation. In the current study, we determined the influence of Mϕ pyroptosis in the host inflammatory response. We found that the levels of TNF-α and IL-1β in PLF are significantly elevated in response to CLP; however, suppressing Mϕ pyroptosis by inhibiting NET formation or deletion of RAGE or caspase-1 markedly decreased the levels of TNF-α and IL-1β in PLF following CLP. In vitro study further showed that pyroptotic Mϕ was able to induce TNF-α and IL-1β expression in normal Mϕ, and the induction is mediated by soluble molecules from pyroptotic cells (Fig. 6c, d), since there is no physical attachment between the pyroptotic Mϕ and the normal Mϕ in the co-culture system

In summary, this study demonstrates a novel mechanism by which NETs through the release of HMGB1 induce Mϕ pyroptosis, which in turn plays an important role in directing the progress of inflammation following infection. These findings shed light on the proinflammatory role of NETs in augmenting inflammation in sepsis, and provide us with new information for generating therapeutic strategy against infectious diseases.

## Materials and methods

### Animal strains

All the mice used in the experiments were 8–10-weeks-old and on a C57BL/6 background. C57BL/6 wild-type (WT) mice were purchased from the Jackson Laboratory (Bar Harbor, ME, USA). TLR4 knockout (TLR4^−/−^) mice, TLR9 knockout (TLR9^−/−^) mice, RAGE knockout (RAGE^−/−^) mice, and HMGB1 knockout (HMGB1^−/−^) mice were obtained from Dr. Timothy Billiar’s laboratory at the University of Pittsburgh. All the animal experimental protocols were reviewed and approved by the Institutional Animal Care and Use Committees of University of Pittsburgh and VA Pittsburgh Healthcare System.

### Reagents

Primary antibodies for cell staining and western blotting: ASC Ab (Santa Cruz Biotechnologies), rabbit polyclonal anti-mouse HMGB1 antibody (Abcam, Cambridge, MA, USA), rabbit polyclonal anti-mouse caspase-1 p10 (Santa Cruz Biotechnologies), and GAPDH (D16H11) XP Rabbit mAb (Cell Signaling Technology). Secondary antibodies including Alexa Fluor 488-conjugated anti-mouse IgG, Cy5-conjugated anti-mouse IgG, Alexa Fluor 488-conjugated anti-rabbit IgG, and Cy3-conjugated anti-rabbit IgG were provided by the Center for Biologic Imaging, University of Pittsburgh Medicine Center. In Situ Cell Death Detection Kit, TMR red (TUNEL) was purchased from Roche (Indianapolis, IN, USA). Annexin-V detection kit was purchased from BD Biosciences. iScript™ Reverse Transcription Supermix and iTaq™ Universal SYBR® Green Supermix were purchased from Bio-Rad. Phorbol 12-myristate 13-acetate(PMA) was from Sigma-Aldrich.

### Mouse model of CLP

The mouse CLP model was carried out, as previously described^20^. Mice were anesthetized with ketamine (50 mg/kg) and xylazine (5 mg/kg) via i.p. injection. After disinfection, a 1 cm midline laparotomy was made in the abdomen. The cecum was then exteriorized, and the distal end was ligated with a 6.0 silk suture and punctured once with a needle (21-gauge) to achieve a sublethal sepsis model. Mice were resuscitated with (5 ml/100 g) saline, and killed at different time points after surgery to retrieve the peritoneal lavage.

### BMDM isolation and culture

The femurs and tibias were harvested from the WT or gene knockout mice, followed by the bone marrow being flushed with prechilled Dulbecco’s modified Eagle's medium (DMEM).^34^ Briefly, the cell pellets were collected by centrifugation at 4 °C, and the erythrocytes were lysed with RBC lysis buffer (Thermo Fisher Scientific). The resultant cells were then washed two times with phosphate-buffered saline (PBS) and suspended in the cell culture medium (DMEM containing 10% fetal bovine serum (FBS) complemented with 50 μg/ml penicillin/streptomycin and 10 ng/ml recombinant macrophage-colony stimulating factor (Sigma-Aldrich, St. Louis, MO, USA)) at a concentration of 1 × 10^6^ cells/ml and seeded into 6-cm ultra-low attachment surface plates (Corning Costar, Corning, NY, USA). The BMDM culture medium was changed on day 3 and day 5. BMDM were entirely differentiated and ready for use at day 7.

### PMNs isolation and NETs induction

PMNs were induced in the peritoneal cavity of the mice, as previously described.^20^ Briefly, mice were injected intraperitoneally with 1 ml 9% casein solution twice overnight. The mice were killed 3 h after the second injection to harvest PLF. PLF was subsequently centrifuged, and the cell pellets were washed. The PMNs were isolated by discontinuous density gradient centrifugation with two commercially available solutions (Histopaque-1077 and Histopaque-1119) of differential density purchased from Sigma (St. Louis, MO), according to the manufacturer’s instructions. PMNs (10^6^ cells/ml) were incubated with 50 nM PMA to induce NET formation.

### NET quantification assay

The PMNs were cultured in 96-well plates at a concentration of 10^6^ cells/ml. At the indicated time points after treatment, 1 U/ml micrococcal nuclease (New England Biolabs, Ipswich, MA) was added. PMNs were incubated at 37 °C for 15 min to allow the extruded DNA to detach from the cell debris. Cells were then centrifuged at 1800 *g* for 10 min. Cell-impermeable DNA-binding dye SYTOX Green (Thermo Fisher Scientific, Waltham, MA) was added to the extracted supernatants and incubated in the dark for 15 min. Extracellular DNA content is represented by the mean fluorescence intensity (MFI) detected with SpectraMax M2 (excitation wavelength 485 nM and emission wavelength 530 nM).

### Western blot

The supernatant of PLF or cell culture medium was concentrated 20 times by Ultracel-3 membrane 3 kDa, and the BMDM lysates were separated by 8% and 15% sodium dodecyl sulfate polyacrylamide gel electrophoresis, and then transferred onto the PVDF membranes. After blocking for 1 h at room temperature with blocking buffer (LI-COR Biosciences, Lincoln, NE, USA), the blots were incubated with the primary antibody at 4 °C overnight, followed by incubation with appropriate secondary antibodies (LI-COR Biosciences) for 1 h. Protein bands were detected using the Odyssey System from LI-COR Biosciences, and the intensity of each band was quantified using ImageJ version 1.50i. The intensity of the target protein band was normalized with a reference protein band and calculated for the fold changing.

### Detection of lysosome rupture and CatB activation

PMϕ/BMDM (5 × 10^5^ cells) cultured in 35 mm Petri dishes were stimulated with NETs (200 μl) for 9 h, followed by co-culturing with DQ Ovalbumin (10 mg/ml; Molecular Probes) or Magic Red CatB assay reagent (10 mg/ml; Immunochemistry Technologies, Bloomington, MN, USA) at 37 °C for 1 h for the detection of lysosome rupture and CatB activation. After fixation with 4% paraformaldehyde, the cells were visualized by confocal microscopy. Cells were randomly selected for the measurement of intracellular fluorescence intensity by using Olympus FV10-ASW software (Olympus).

### Flow cytometry analysis of programmed cell death and pyroptosis

Programmed cell death was analyzed by flow cytometry with apoptosis detection kit (BD Biosciences, Franklin Lakes, NJ). The peritoneal macrophages (PMs)/BMDM were centrifuged, washed twice with prechilled PBS, and resuspended in the binding buffer. PMs/BMDM were incubated with Annexin-V and 7-AAD for 15 min at room temperature in the dark, and then were analyzed by flow cytometry. The cells double-stained positive for Annexin-V and 7-AAD were considered to be programmed cell death. Cell pyroptosis was detected by two-color flow cytometry. PMs or BMDM were incubated with Alexa Fluor 488- labeled caspase-1 FLICA at 37 °C for 1 h. After being fixed with 4% paraformaldehyde, the PMs/BMDM were stained with TMR red-labeled In-Situ Cell Death Detection reagent (Roche Applied Science, Indianapolis, IN, USA), following the manufacturer’s instructions. The cells were then analyzed by flow cytometry. The double-stained cells were identified as pyroptotic cells. Background and auto-fluorescence were determined by a control antibody with the same isotype staining. Acquisition was performed on 10,000 events using a FACScalibur cytometer (BD Biosciences, San Jose, CA, USA), BD LSR II (BD Biosciences) and FlowJo-V10 software (Tree Star, Ashland, OR, USA). The rate of cell death was calculated as (dead cells/total cells) × 100%.

### Mϕ–Mϕ co-incubation

Mϕ–Mϕ co-incubation was performed using Transwell plates (Corning Incorporated Life Sciences, Acton, MA, USA). BMDM (5 × 10^5^ cells per well) were plated and cultured in the top well of the Transwell, then treated with NETs for 18 h to induce pyroptosis, followed by transferring from the top well into a new Transwell where untreated BMDM were cultured in the bottom well. The co-cultures were then incubated for 6 h in DMEM containing 10% FBS.

### RNA extraction and quantitative real-time PCR

The BMDM from the co-culture bottom wells were harvested, and total RNA was isolated by using TRIzol RNA Isolation Reagent (Thermo Fisher Scientific), following the manufacturer’s instruction. Quantitative real-time PCR was done using iTaq Universal SYBR Green Supermix (1725121, Bio-Rad) in a Bio-Rad iQ5 real-time PCR machine (Bio-Rad). The specific primers for mouse TNF-α and IL-1β were also purchased from Bio-Rad: TNF-α forward, 5′-GACGTGGAACTGGCAGAAGAG-3′ and reverse, 5′-TTGGTGGTTTGTGAGTGTGAG-3′; IL-1β forward, 5′-GAAATGCCACCTTTTGACATG-3′ and reverse, 5′-TGGATGCTCTCATCAGGACAG-3′. After the amplification protocol was over, PCR product was subjected to melt curve analysis using Bio-Rad iQ5 software. The fold change was calculated using the ΔΔ*C*_T_ threshold cycle method^35^ and the value for the GAPDH gene, which was normalized to untreated groups.

### Data presentation and statistical analysis

The data are presented as mean ± S.E.M. of the indicated number of experiments. SPSS 19.0 was used for statistical analysis. Significances between groups were determined by using one-way ANOVA or two-tailed Student’s *t*-test. *P* < .05 was considered as statistically significant.
